# AKT-AMPKα-mTOR-dependent HIF-1α Activation is a New Therapeutic Target for Cancer Treatment: A Novel Approach to Repositioning the Antidiabetic Drug Sitagliptin for the Management of Hepatocellular Carcinoma

**DOI:** 10.3389/fphar.2021.720173

**Published:** 2022-01-12

**Authors:** Eslam E. Abd El-Fattah, Sameh Saber, Mahmoud E. Youssef, Hanan Eissa, Eman El-Ahwany, Noha A. Amin, Mohammed Alqarni, Gaber El-Saber Batiha, Ahmad J. Obaidullah, Mohamed M.Y. Kaddah, Ahmed Gaafar Ahmed Gaafar, Ahmed A.E. Mourad, Gomaa Mostafa-Hedeab, Amir Mohamed Abdelhamid

**Affiliations:** ^1^ Department of Biochemistry, Faculty of Pharmacy, Delta University for Science and Technology, Gamasa, Egypt; ^2^ Department of Pharmacology, Faculty of Pharmacy, Delta University for Science and Technology, Gamasa, Egypt; ^3^ Department of Clinical Pharmacology, Faculty of Medicine, Mansoura University, Mansoura, Egypt; ^4^ Department of Immunology, Theodor Bilharz Research Institute, Giza, Egypt; ^5^ Department of Hematology, Theodor Bilharz Research Institute, Giza, Egypt; ^6^ Department of Pharmaceutical Chemistry, College of Pharmacy, Taif University, Taif, Saudi Arabia; ^7^ Department of Pharmacology and Therapeutics, Faculty of Veterinary Medicine, Damanhour University, Damanhour, Egypt; ^8^ Drug Exploration and Development Chair (DEDC), Department of Pharmaceutical Chemistry, College of Pharmacy, King Saud University, Riyadh, Saudi Arabia; ^9^ Department of Pharmaceutical Chemistry, College of Pharmacy, King Saud University, Riyadh, Saudi Arabia; ^10^ Pharmaceutical and Fermentation Industries Development Center, City of Scientific Research and Technological Applications, New Borg El-Arab, Egypt; ^11^ Department of Pharmacology and Toxicology, Faculty of Pharmacy, Port Said University, Port Said, Egypt; ^12^ Pharmacology Department and Health Research Unit, Medical College, Jouf University, Jouf, Saudi Arabia; ^13^ Pharmacology Department, Faculty of Medicine, Beni-Suef University, Beni Suef, Egypt

**Keywords:** hepatocellular carcinoma, sitagliptin, HIF-1α, Akt, AMPKα, mTOR, MAPK, angiogenesis

## Abstract

HIF-1α is a key factor promoting the development of hepatocellular carcinoma (HCC). As well, AKT-AMPKα-mTOR signaling is a promising target for cancer therapy. Yet, the AKT-AMPKα-mTOR-dependent activation of HIF-1α has not been studied in livers with HCC. In addition, the mechanisms underlying the potential antineoplastic effects of sitagliptin (STGPT), an antidiabetic agent, have not yet been elucidated. For that purpose, the N-nitrosodiethylamine (NDEA)-induced HCC mouse model was used in the present study using a dose of 100 mg/kg/week, i.p., for 8 weeks. NDEA-induced HCC mice received STGPT 20, 40, or 80 mg/kg starting on day 61 up to day 120. The present study revealed that STGPT inhibited HIF-1α activation via the interference with the AKT-AMPKα-mTOR axis and the interruption of IKKβ, P38α, and ERK1/2 signals as well. Accordingly, STGPT prolonged the survival, restored the histological features and improved liver function. Additionally, STGPT inhibited angiogenesis, as revealed by a significant downregulation in the VEGF and mRNA expression of CD309 with concomitant inhibition of tissue invasion was evident by an increased ratio of TIMP-1/MMP-2. STGPT exhibited apoptotic stimulatory effect as indicated upon calculating the BCL-2/Bax ratio and by the gene expression of p53. The decrease in AFP and liver index calculation, gene expression of Ki-67 confirmed the antiproliferative activity of STGPT. The anti-inflammatory potential was revealed by the decreased TNF-α level and the downregulation of MCP-1 gene expression. Moreover, an antifibrotic potential was supported by lower levels of TGF-β. These effects appear to be GLP1R-independent. The present study provides a potential basis for repurposing STGPT for the inhibition of HCC progression. Since STGPT is unlikely to cause hypoglycemia, it may be promising as monotherapy or adjuvant therapy to treat diabetic or even normoglycemic patients with HCC.

## Introduction

Hepatocellular carcinoma (HCC), the most common form of primary liver cancer, ranked sixth in incidence and fourth in mortality, with 841,080 new cases and 781,631 deaths worldwide (Ko et al., 2020). Over the past three decades, the global incidence of liver cancer has risen by 75%. Hepatitis B and C virus infection and cirrhosis are among the risk factors for HCC (Singal et al., 2020). HCC typically arises in a background of chronic liver disease and is often discovered at later stages, making treatment choices more complex. In addition, the HCC needs to be treated, but the underlying liver disease will often require medical attention. Therefore, the management of HCC requires a multidisciplinary approach, including the gastroenterologist, oncologist, interventional radiologist, and surgeon.

First-line therapy for late-stage HCC includes sorafenib and lenvatinib. However, such systemic chemotherapies have proved disappointing because they provide an extremely low survival rate in newly diagnosed patients with HCC. Besides, they cannot specifically target cancer cells that lead to a wide array of side effects. Patients on these drugs develop resistance within a few months and have to rely on second-line therapy that includes regorafenib, pembrolizumab, nivolumab, and cabometyx.

It was reported that diabetes mellitus (DM) increased the risk of HCC only in the presence of other risk factors in a study that included 823 HCC cases and 3459 controls (El-Serag et al., 2001). Also, it was discovered that diabetes increased the risk of developing HCC in both univariate and multivariable analyses (Yang et al., 2020). Additionally, in HCV-infected patients, DM increased the risk of developing HCC (Huang et al., 2017). Further, Arboatti et al. (2019) stated that high-fat diet-induced type II DM sensitizes mice to N-nitrosodiethylamine (NDEA)-induced HCC.

AMP-activated protein kinase (AMPK) is a metabolic enzyme that serves as a eukaryotic cellular energy sensor and plays a vital role in cell growth and metabolism coordination, making it a promising target for cancer therapy (Saber et al., 2020a). In many animal cancer models, pharmacologic activators of AMPK, such as metformin, phenformin, AICAR, and A769662, prevented or delayed the onset of tumorigenesis (Hawley et al., 2012; Yang et al., 2012; Saber et al., 2020b). Population study also showed that metformin therapy is associated with reduced risk in HCC patients with type II DM and seems to have a protective effect on HCC development (Donadon et al., 2010; Chen et al., 2013).

Sitagliptin (STGPT), an antidiabetic dipeptidyl peptidase 4 (DPP4) inhibitor, prolongs the effect of the incretin hormone, glucagon-like peptide 1 (GLP-1), and glucose-dependent insulinotropic polypeptide (GIP). It stimulates insulin release and lowers glucagon secretion. DPP4 inhibitors improve the survival rate in patients with prostate cancer; however, not in patients with pancreatic or breast cancer (Shah et al., 2020). STGPT showed potential anticancer activities against gastric cancer by inhibiting the AMPK/YAP/melanoma-associated antigen-A3 pathway (Wang et al., 2020). One report proposed that a one-year use of STGPT may reduce the risk of breast cancer in female diabetic patients (Tseng, 2017). Another report revealed that STGPT inhibited the progression of HCC by activating lymphocyte chemotaxis in the clinical setting (Nishina et al., 2018). Moreover, Jiang et al. (2018) found that STGPT (10 and 20 mg/kg) has a potential protective effect against the NDEA-induced liver cancer by inhibiting inflammation and NF-κB activation. However, we believe that the mechanisms undelaying the antitumor activity of STGPT may go beyond the interference with NF-κB signaling.

A second mediator that promotes HCC progression is hypoxia-inducible facto-1α (HIF-1α). This transcription factor senses the intratumoral oxygen tension and subsequently mediates the activation of hypoxia response; accordingly, HIF-1α can serve as a potential anticancer target. It was suggested that inhibition of HIF-1α transcriptional activation and expression in HCC cells could suppress angiogenesis and enhance apoptosis (Zhou et al., 2020). A connection between AMPK and the master regulator of hypoxic adaptation via gene transcription, HIF1-α, has also been considered (Dengler, 2020). Therefore, we suggest that AMPK activators might modulate AMPK/HIF-1α crosstalk in hypoxic conditions. The characterized hypoxia region inside the HCC tumors has been recently found as the key driver of HCC malignance and treatment failure, leading to various hypoxia-related biological consequences, including angiogenesis, metastasis, metabolism deregulation, and drug resistance, ultimately resulting in treatment failure.

Despite all of the progress in developing targeted therapeutic approaches for the management of HCC; however, STGPT could reduce disease mortality as early as possible. STGPT repositioning uncovers such an avenue of investigation in a rapid manner and at a reduced cost. The present study aimed to elucidate the mechanisms underlying the potential antitumor activity of STGPT with a particular emphasis on studying the AMPK/HIF-1α interactions as a novel therapeutic target for managing HCC. We also investigated the involvement of the NF-κB and MAPK signaling in HIF-1α regulation. Additionally, examination of the apoptosis and autophagy state of the NDEA-intoxicated liver in response to STGPT was also considered. Further, beneficial effects against angiogenesis, proliferation, and metastasis were explored. Investigations were carried out using the NDEA-intoxicated mouse as a well-recognized model of HCC (Saber et al., 2018a; Saber et al., 2018b; Saber et al., 2019; Younis et al., 2019).

## Materials and Methods

### Animals

Adult male CD-1 Swiss albino mice weighing 20 ± 2 g were purchased from the animal facility of the Faculty of Pharmacy, Delta University for Science and Technology. The mice were fed standard rodent chow, permitted ad libitum access to water. Standard environmental conditions were maintained (21°C, 45–55% humidity and light: dark cycles 12:12 h). Animals were allowed an acclimatization period of 1 week prior to initiating the protocol. Mice were treated and sacrificed following the relevant guidelines of the Delta University for Science and Technology, IACUC approval number (FPDUST23121/2).

### Experimental Design

After the acclimatization phase, mice were randomly divided into six groups as follows: Control (*n* = 10), in which mice administered intraperitoneal injection (i.p.) of normal saline solution as the vehicle of NDEA; STGPT (*n* = 10), in which mice administered normal saline solution (i.p.) as the vehicle of NDEA + STGPT (80 mg/kg/day, p.o.) starting on the day 61 of the experiment and up to the day 120; NDEA (*n* = 15), in which mice administered NDEA (100 mg/kg/week, i.p.) for 8 weeks; NDEA + STGPT 20 (*n* = 15), in which mice administered NDEA (100 mg/kg/week, i.p.) for 8 weeks + STGPT (20 mg/kg/day, p.o.) starting on the day 61 up to the day 120; NDEA + STGPT 40 (*n* = 15), in which mice administered NDEA (100 mg/kg/week, i.p.) for 8 weeks + STGPT (40 mg/kg/day, p.o.) starting on the day 61 up to the day 120; NDEA + STGPT 80 (*n* = 15), in which mice administered NDEA (100 mg/kg/week, i.p.) for 8weeks + STGPT (80 mg/kg/day, p.o.) starting on the day 61 up to the day 120. Mice were anesthetized by thiopental sodium (20 mg/kg) at the end of the experiment (120 days), sacrificed, and their livers were dissected and weighed. After collecting blood, sera were separated and preserved for biochemical investigations at −80°C. Using ice-cold saline, fresh livers were washed and dried on a clean paper towel. Each liver was separated into two portions; one portion for histopathological analysis preserved in 4% formalin for 24 h; the second portion was immediately frozen in liquid nitrogen and kept at -80°C for qRT-PCR, ELISA, and colorimetric assays.

### Calculation of the Liver Index

The liver index was calculated as the liver weight (g) divided by the bodyweight (g).

### Histopathological Examination

Hepatic samples were fixed in 4% neutral-buffered formalin under a fume hood for 24 h. Tissue samples were removed from the fixation jar and fit into cassettes. Samples were dehydrated by the addition of serial dilutions of ethanol (70%-95%–100%). Paraffin solution was added to cassettes, and samples were retrieved and kept overnight. After infiltration of tissue samples with paraffin solution, they were removed and positioned in a specific metallic base mold in the correct orientation, then melted paraffin was added and placed immediately on the cooling surface. Paraffin blocks were cut into sections with 5-µm thickness using a microtome. Sections were dewaxed by xylene then rehydrated by adding 100 and 95% ethanol for 5 min. Dewaxed sections were stained with hematoxylin and eosin (H&E) for histopathological examination and necroinflammation scoring. A pathologist blindly examined sections.

### Assessment of Necroinflammation

For the evaluation of the intensity of necroinflammation, the sum of the following scoring criteria was applied: periportal or peri septal interface hepatitis (0–4); confluent necrosis (0–6); spotty lytic necrosis, apoptosis, and focal inflammation (0–4); and portal inflammation (0–4) (Abdelhamid et al., 2020; Khalil et al., 2020; Abdelhamid et al., 2021).

### Biochemical Analysis

#### Assessment of Oxidative Stress and Liver Function

Alanine transaminase (ALT, Cat. No. AL1031), aspartate transaminase (AST, Cat. No. AS1061), alkaline phosphatase (ALP, Cat. No. AP1020), and total antioxidant capacity (TAC, Cat. No. TA 2513) were measured spectrophotometrically in serum using commercial kits (Bio-diagnostic, Giza, Egypt). Gamma-glutamyltransferase (γGT, Cat. No. MAK089) was measured spectrophotometrically in serum using commercial kits (Sigma-Aldrich, St. Louis, MO, United States).

#### Assessment of AFP, TNF-α, VEGF, and MMP-2

After rinsing in PBS to remove blood, wet liver tissue (100 mg) was homogenized with 500 μL of lysis buffer, incubated on ice for 20 min, then centrifuged at 18,000 x g for 20 min at 4°C. The supernatants were transferred into clean tubes, and pellets were discarded. The sample protein concentration in the extract was determined using a BCA protein assay reagent kit purchased from Thermo Fisher Scientific Inc., and samples were diluted to the desired concentration then stored at -80°C. According to the manufacturer’s instructions and using Quantikine^®^ ELISA assay kits (R&D Systems, Minneapolis, MN, United States), Tumor necrosis factor-alpha (TNF-α, Cat. No. MTA00B), vascular endothelial growth factor (VEGF, Cat. No. MMV00), matrix metalloproteinase-2 (MMP-2, Cat. No. MMP200) and serum alpha-fetoprotein (AFP, Cat. No. MAFP00) were quantified.

#### Assessment of TGF-β, BCL-2, TIMP-1, BCL-2, Bax, and ULK1

Tissue inhibitor of metalloproteinase-1 (TIMP-1, Cat. No. LS-F26009) and B-cell lymphoma 2 (BCL-2, Cat. No. CSB-E08855m) concentrations were measured using CUSABIO ELISA assay kits (MyBioSource Inc., San Diego, CA, United States) with proper recommended protocol. TIMP-1 was measured by ELISA using commercial kits obtained from LSBio, Inc. (Germany, Cat. No.LS-F26009). The UNC-51-like kinase 1 level (ULK1, Cat. No. MBS9326629) was assessed using ULK1 ELISA assay kits (MyBioSource Inc.). BCL2-associated X protein (Bax, Cat. No. MBS2607437) and transforming growth factor-beta (TGF-β, Cat. No. MBS824944) were measured using an ELISA kit obtained from MyBioSource Inc.

#### Assessment of Hypoxia-Inducible Factor 1-Alpha (HIF-1α)

The sample protein concentration in the extract was determined using a BCA protein assay reagent kit purchased from Thermo Fisher Scientific Inc. (Rockford, United States), and samples were measured using ELISA commercial kits (Abcam, Cambridge, MA, United States; Cat. No. ab275103).

#### Assessment of Phospho-Protein Kinase B (p-AKT) (Ser473)/AKT Ratio and Phospho-5′ AMP-Activated Protein Kinase (p-AMPK)/AMPK Ratio

p-AKT (Ser473)/AKT was measured by ELISA using commercial kits obtained from Abcam (Cat. No. ab126433). Using ELISA commercial kits obtained from RayBiotech (Norcross, GA, Cat. No. PEL-AMPKA-S487-T-1), p-AMPK/AMPK concentration was measured; After pipetting samples into the wells, AMPKa1 present in the sample was bound to the wells by the immobilized antibody, and wells were washed. In select wells, a rabbit anti-phospho-AMPKa1 (S487) antibody was added to detect phosphorylated AMPKa1. In the remaining wells, pan AMPKa1 was assessed using a biotinylated antibody for pan-AMPKa1. After washing, HRP-Streptavidin was pipetted into the wells, washed, and then TMB substrate solution was added to the wells.

#### Assessment of p-mTOR (Ser2448), p38 MAPK (pT180/Y182), and ERK1/2 (pT202/Y204)

Using the manufacturer’s protocol, the phospho-mechanistic target of rapamycin (p-mTOR) (Ser2448, Cat. No. ab168538), phospho-mitogen-activated protein kinase p38 (p38 MAPKα) (pT180/Y182, Cat. No. ab221011), and phospho-extracellular regulated kinase (p-ERK1/2) (pT202/Y204, Cat. No. ab176640) were measured by ELISA kits obtained from Abcam**.**

#### Assessment of the Phospho-Inhibitor κB Kinase Complex p-IκBκB (Ser177/181)

A PathScan sandwich ELISA kit was used to measure the endogenous levels of IκBκB (pSer177/181) following the manufacturer’s protocol (PathScan, Cell Signaling Technology, Danvers, MA, United States, Cat. No. 7080).

#### Quantitative Real-Time PCR for the Expression of CD309, Monocyte Chemoattractant Protein-1 (MCP-1), Forkhead Box O3 (FOXO3), P53, Ki-67, and GLP1R in Liver Tissue

Total RNA was extracted from liver tissue using RNeasy Mini kit (QIAGEN, Germany) and then reverse-transcribed into cDNA using Quantiscript reverse transcriptase (QuantiTect Reverse Transcription Kit, QIAGEN, Germany). The target gene was amplified in SYBR Green PCR Master Mix (Yeasen Biotech, China) with a specific primer by thermocycler Rotor-Gene Q (Qiagen, Hilden, Germany) (96-well 0.2-ml Block). The relative expression of target gene mRNA was normalized to GAPDH and calculated using the 2^–ΔΔCt^ method. Three replicates were set for each gene, and the primer sequences of the detected genes are listed in Table 1.

**TABLE 1:** Primer sequences for qRT-PCR.

Primer	GenBank accession	F 5'->3′	R 5'->3′	Amplicon size (bp)
MCP-1	NM_011333.3	TCA​GCC​AGA​TGC​AGT​TAA​CGC	TCT​GGA​CCC​ATT​CCT​TCT​TGG	185
CD309	NM_001363216.1	GAG​AGC​AAG​GCG​CTG​CTA​GC	GAC​AGA​GGC​GAT​GAA​TGG​TG	390
FOXO3	NM_001376967.1	AGC​CGT​GTA​CTG​TGG​AGC​TT	TCT​TGG​CGG​TAT​ATG​GGA​AG	180
P53	NM_001127233.1	TGA​AAC​GCC​GAC​CTA​TCC​TTA	GGC​ACA​AAC​ACG​AAC​CTC​AAA	92
Ki-67	NM_001081117.2	CTG​CCT​CAG​ATG​GCT​CAA​AGA	GAA​GAC​TTC​GGT​TCC​CTG​TAA​C	151
GLP1R	NM_021332.2	TGA​ACC​TGT​TTG​CAT​CCT​TCA	ACT​TGG​CAA​GCC​TGC​ATT​TGA	513
GAPDH	NM_001289726.1	TCA​AGA​AGG​TGG​TGA​AGC​AG	AGG​TGG​AAG​AAT​GGG​AGT​TG	111

#### Statistical Analysis

Statistical analysis was conducted using GraphPad Prism software version 8.0.2 (GraphPad Software Inc., La Jolla, CA, United States). The Mantel-Cox test was carried out for survival rate analysis to determine the significance of between-group differences in the Kaplan–Meier survival analysis. Necroinflammatory scores are presented as the median with interquartile range and were compared using Kruskal-Wallis followed by Dunn’s multiple comparison test. Parametric data are presented as the mean ± standard deviation (SD), and differences between groups were analyzed by one-way analysis of variance followed by Tukey’s Kramer multiple comparison test. Significance was accepted at *p* < 0.05.

## Results

### STGPT Prolonged the Survival and Decreased Liver Index in NDEA-Administered Mice

**FIGURE 1:**
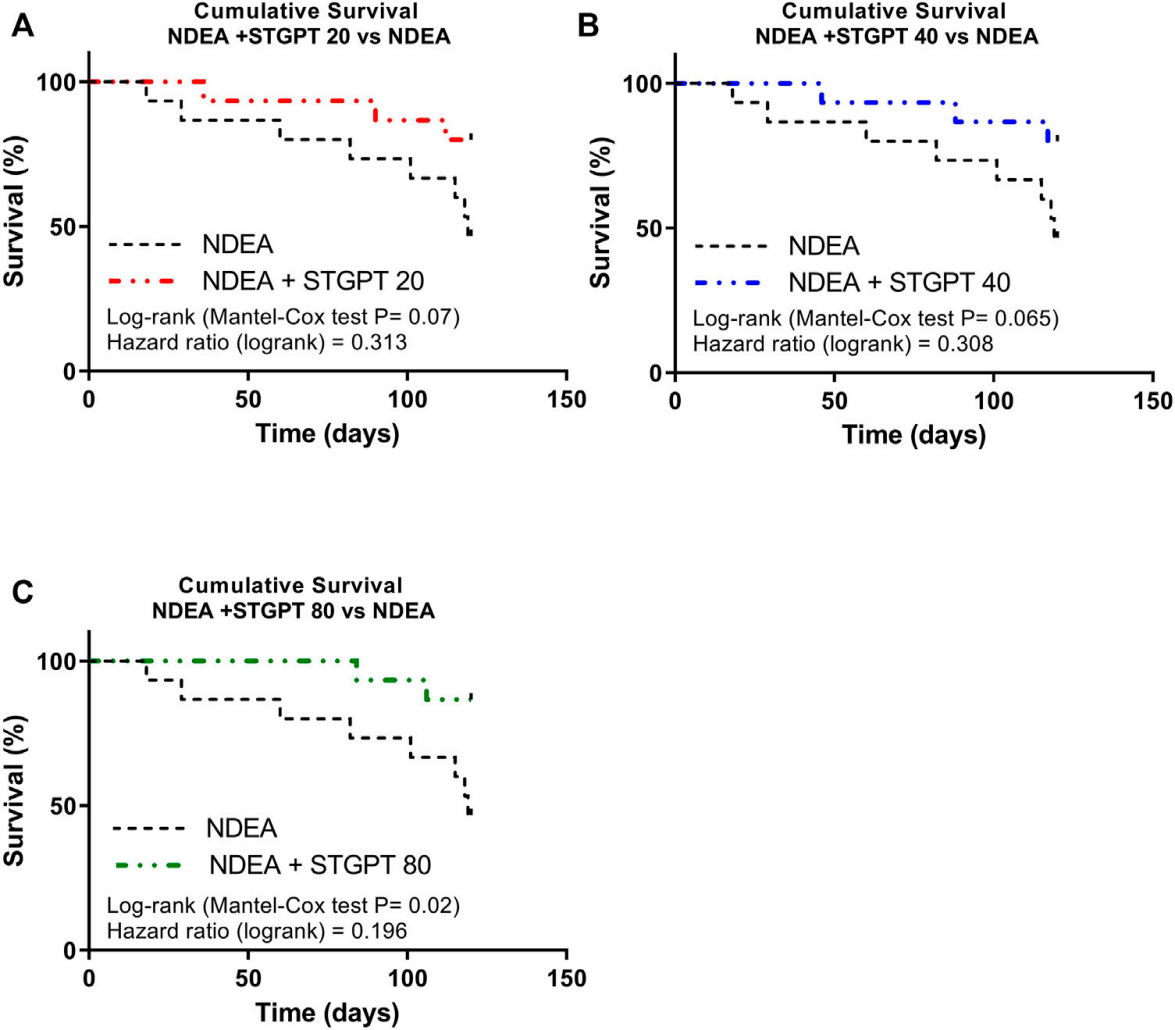
Effect of STGPT (20, 40, and 80 mg/ kg) on survival rate **(A,B,C)** in mice with NDEA-induced HCC. Control, normal control group received the vehicle; STGPT 80, normal group received sitagliptin (80 mg/ kg); NDEA, NDEA-induced HCC group received the vehicle; NDEA + STGPT 20, NDEA-induced HCC group treated with sitagliptin (20 mg/kg); NDEA + STGPT 40, NDEA-induced HCC group treated with sitagliptin (40 mg/kg); NDEA + STGPT 80, NDEA-induced HCC group treated with sitagliptin (80 mg/kg). Data in figure (d) are presented as the mean ± SD (n = 6). ^++++^ *p* < 0.0001 *vs.* Control group, ****p < 0.0001 *vs.* NDEA group.

The survival analysis for the effect of STGPT on mice with NDEA-induced HCC revealed that oral administration of STGPT (80 mg/kg) significantly increased the cumulative survival percent of NDEA-administered mice **(** Figure 1C). In contrast, treatment with STGPT 20 mg/kg (Figure 1A) or 40 mg/kg (Figure 1B) resulted in a non-significant increase in survival percent compared to the NDEA group. There was also a marked increase in liver weight and the liver index in the NDEA group compared to the control group, while the STGPT 20, 40, and 80 groups showed a significant reduction in their liver weights and indices compared to that of the NDEA group (Table 2)**.**

**TABLE 2:** Effect of STGPT (20, 40 and 80 mg/kg) on liver weight, body weight and liver index in mice with NDEA-induced HCC.

Animal group	Liver weight (g)		Body weight (g)		Liver index (liver weight/Body weight)	
Control	1.06 ± 0.15		36.93 ± 2.14		0.02895 ± 0.006	
STGPT 80	1.09 ± 0.16		36.06 ± 3.36		0.03072 ± 0.007	
NDEA	1.82 ± 0.19	++++	27.48 ± 3.39	++++	0.06752 ± 0.014	++++
NDEA + STGPT 20	1.28 ± 0.18	****	30.25 ± 2.06	++	0.04225 ± 0.006	****
NDEA + STGPT 40	1.21 ± 0.14	****	28.66 ± 1.84	+++	0.04206 ± 0.004	****
NDEA + STGPT 80	1.07 ± 0.11	****	32.12 ± 1.82	*	0.03353 ± 0.003	****
				+		

Data are presented as the mean ± SD (*n* = 6). +*p* < 0.05 *vs*. Control group, ++*p* < 0.01 *vs*. Control group, +++*p* < 0.001 vs Control group, ++++*p* < 0.0001 *vs*. Control group, *p < 0.05 *vs*. NDEA group, ****p < 0.0001 vs NDEA group. Control, normal control group received the vehicle; STGPT 80, normal group received sitagliptin (80 mg/kg); NDEA, NDEA-induced HCC group received the vehicle; NDEA + STGPT 20, NDEA-induced HCC group treated with sitagliptin (20 mg/kg); NDEA + STGPT 40, NDEA-induced HCC group treated with sitagliptin (40 mg/kg); NDEA + STGPT 80, NDEA-induced HCC group treated with sitagliptin (80 mg/kg).

### STGPT Ameliorated Histopathological Characteristics and Improved Necroinflammatory Score in NDEA-Administered Mice

**FIGURE 2:**
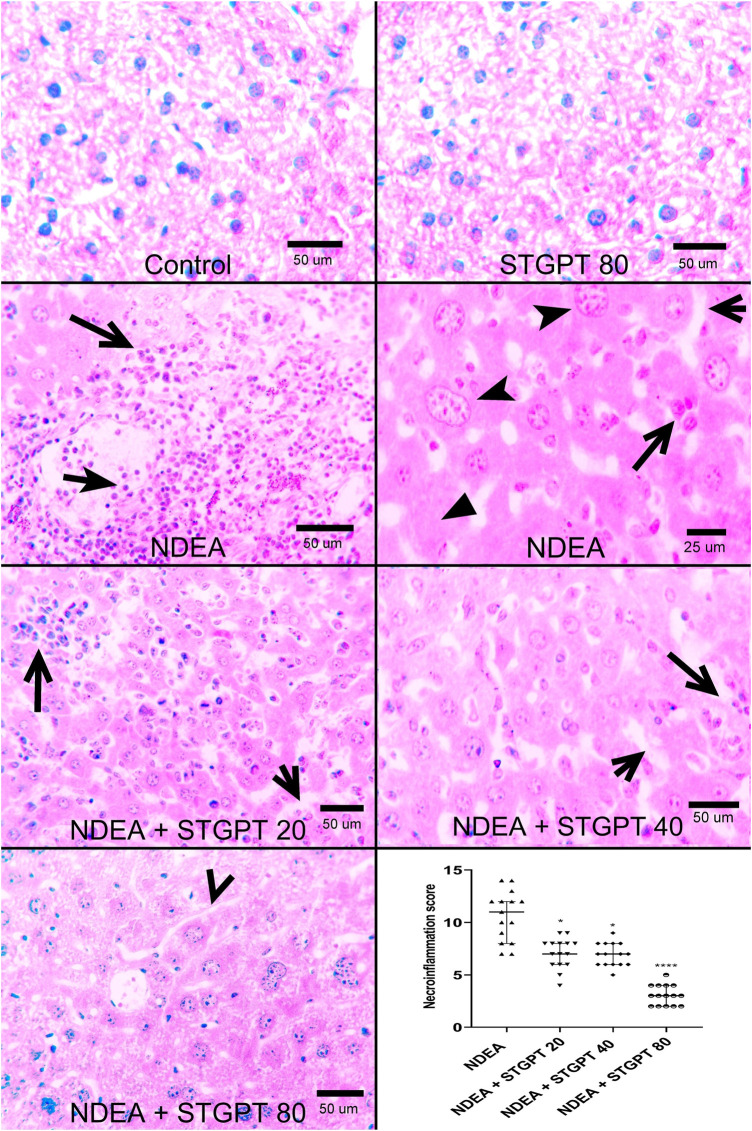
Effect of STGPT (20, 40, and 80 mg/kg) on histopathological characteristics and necroinflammatory score in mice with NDEA-induced HCC. Representative histological appearance of liver tissue specimens from Control and STGPT 80 showing normal hepatic architecture; Liver sections from NDEA group (left and right panels) showing disrupted hepatic architecture due to the disorganization of hepatic cords, dilatation of hepatic sinusoids (short open arrow) with inflammatory cell infiltration (long open arrows), spotty necrosis (filled arrowhead) and a great number of hepatocytes having large nuclei with prominent and multiple nucleoli (notched arrowhead); Liver sections from NDEA + STGPT 20 and NDEA + STGPT 40 (left panel and right panel, respectively) showing mild disruption of hepatic architecture with dilated sinusoids (short open arrow), decreased intralobular leukocyte infiltration (long open arrow), small number of hepatocytes having large nuclei; Liver sections from NDEA + STGPT 80 showing normal hepatic sinusoids (open arrowhead), decreased disorganization and restored hepatic architecture, very mild intralobular leukocyte infiltration, and diminished hepatocytes having large nuclei. H&E, Bar = as indicated. Calculation of the necroinflammation score reveals that Control and STGPT 80 groups show a score of zero and that treatment groups, particularly the NDEA + STGPT 80, significantly decreased the necroinflammation score compared with that of the NDEA-treated group of rats. Data are presented as the median ± interquartile range (*n* = 6). ^+^ *p* < 0.05 *vs.* Control group, ^++^ *p* < 0.01 *vs.* Control group, ^+++^ *p* < 0.001 *vs.* Control group, ^++++^ *p* < 0.0001 *vs.* Control group, *p < 0.05 *vs.* NDEA group, **p < 0.01 *vs.* NDEA group, ***p < 0.001 *vs.* NDEA group, ****p < 0.0001 vs NDEA group, ^#^ *p* < 0.05 vs STGPT 20 group, ^##^ *p* < 0.*vs.* STGPT 20 group, ^###^ *p* < 0.001 *vs.* STGPT 20 group, ^####^ *p* < 0.0001 vs STGPT 20 group, ^@^ *p* < 0.05 *vs.* STGPT 40 group, ^@@^ *p* < 0.01 *vs.* STGPT 40 group, ^@@@^ *p* < 0.001 *vs.* STGPT 40 group, ^@@@@^ *p* < 0.0001 *vs.* STGPT 40 group. Control, normal control group received the vehicle; STGPT 80, normal group received sitagliptin (80 mg/kg); NDEA, NDEA-induced HCC group received the vehicle; NDEA + STGPT 20, NDEA-induced HCC group treated with sitagliptin (20 mg/kg); NDEA + STGPT 40, NDEA-induced HCC group treated with sitagliptin (40 mg/kg); NDEA + STGPT 80, NDEA-induced HCC group treated with sitagliptin (80 mg/kg).

Photomicrograph of liver specimens stained with H&E (Figure 2) from the Control and STGPT 80 groups showed standard hepatic architecture. In comparison, the NDEA group showed disrupted architecture with loss of lobular pattern, dilatation of hepatic sinusoids with inflammatory cell infiltration, spotty necrosis, and many hepatocytes with nuclear anaplasia (mega nuclei). Upon treatment with STGPT (20 and 40 mg/kg), the NDEA-intoxicated mice showed a moderate restoration of architecture. A small number of hepatocytes had large nuclei, and a reduction in the inflammatory-cell infiltration was observed. Moreover, treatment with STGPT 80 mg/kg resulted in a marked regression of malignant changes with very mild disruption of hepatic architecture, decreased intralobular-leukocyte infiltration with a significant reduction in the necroinflammatory score.

### STGPT Decreased ALT, AST, ALP, and γGT and Increased TAC in NDEA-Administered Mice

**FIGURE 3:**
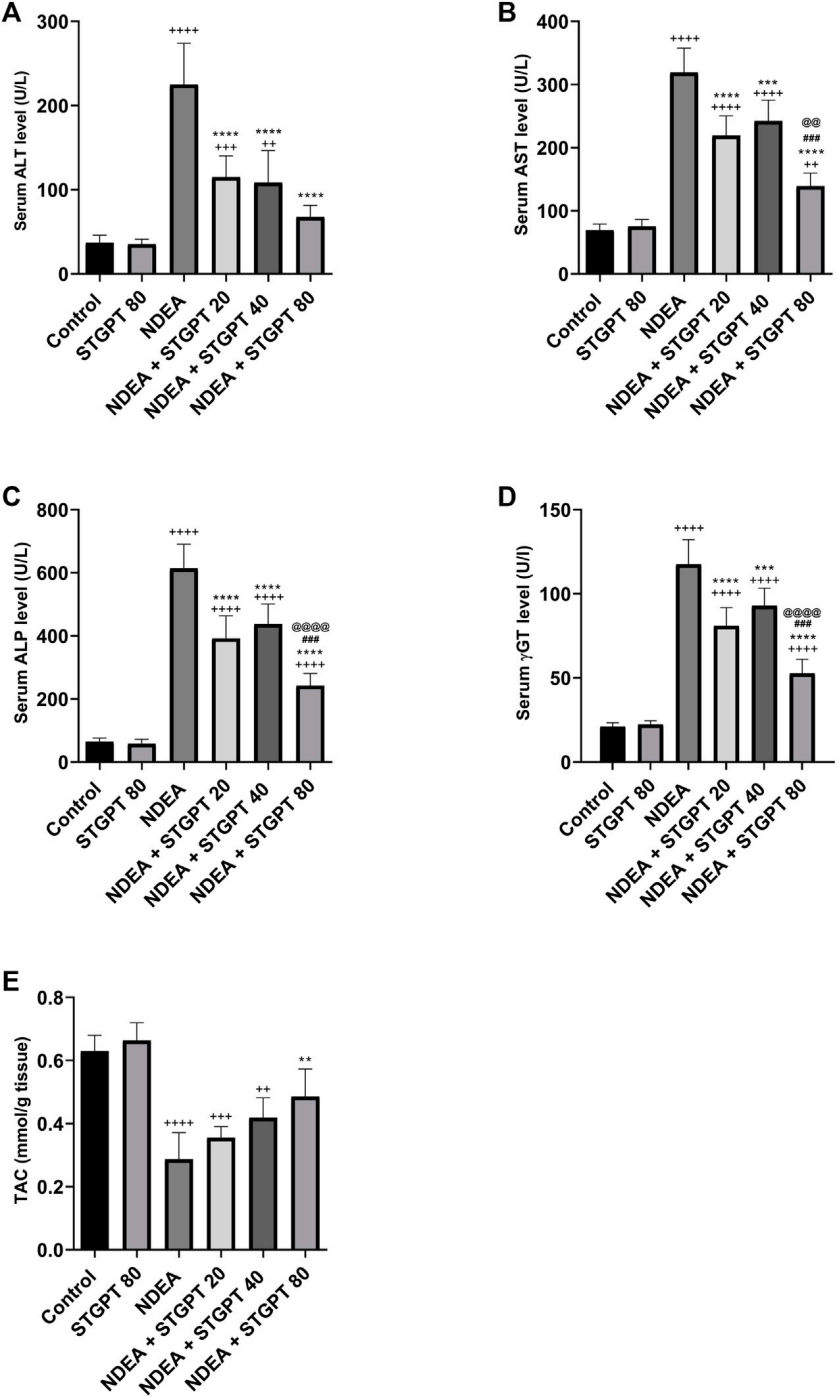
Effect of STGPT (20, 40 and 80 mg/kg) on ALT **(A)**; AST **(B)**; ALP **(C)**; γ-GT **(D)**; and TAC **(E)** in mice with NDEA-induced HCC. Data are presented as the mean ± SD (*n* = 6). ^+^ *p* < 0.05 *vs.* Control group, ^++^ *p* < 0.01 *vs.* Control group, ^+++^ *p* < 0.001 *vs.* Control group, ^++++^ *p* < 0.0001 *vs.* Control group, **p < 0.01 vs NDEA group, ***p < 0.001 *vs.* NDEA group, ****p < 0.0001 *vs.* NDEA group, ^###^ *p* < 0.001 vs STGPT 20 group, ^@@^ *p* < 0.01 vs STGPT 40 group, ^@@@@^ *p* < 0.0001 vs STGPT 40 group. Control, normal control group received the vehicle; STGPT 80, normal group received sitagliptin (80 mg/kg); NDEA, NDEA-induced HCC group received the vehicle; NDEA + STGPT 20, NDEA-induced HCC group treated with sitagliptin (20 mg/kg); NDEA + STGPT 40, NDEA-induced HCC group treated with sitagliptin (40 mg/kg); NDEA + STGPT 80, NDEA-induced HCC group treated with sitagliptin (80 mg/kg).

When compared to the control group, NDEA administration significantly increased serum levels of ALT (Figure 3A), AST (Figure 3B), ALP (Figure 3C), and γGT (Figure 3D), indicating liver damage. In addition, when compared to the control group, it significantly reduced the hepatic tissue level of TAC (Figure 3E). Treatment with STGPT 20 or 40 mg/kg, on the other hand, significantly reduced these elevated levels of ALT, AST, ALP, and γ -GT and increased the hepatic TAC when compared to the NDEA group. The use of STGPT 80 mg/kg resulted in a more significant improvement in these markers than STGPT 20 and 40 mg/kg when compared to the NDEA group; it also normalized the serum level of ALT and TAC content.

### STGPT Decreased the Tissue Content of TNF-α, TGF-β, and Serum Level of AFP in NDEA-Administered Mice

**FIGURE 4:**
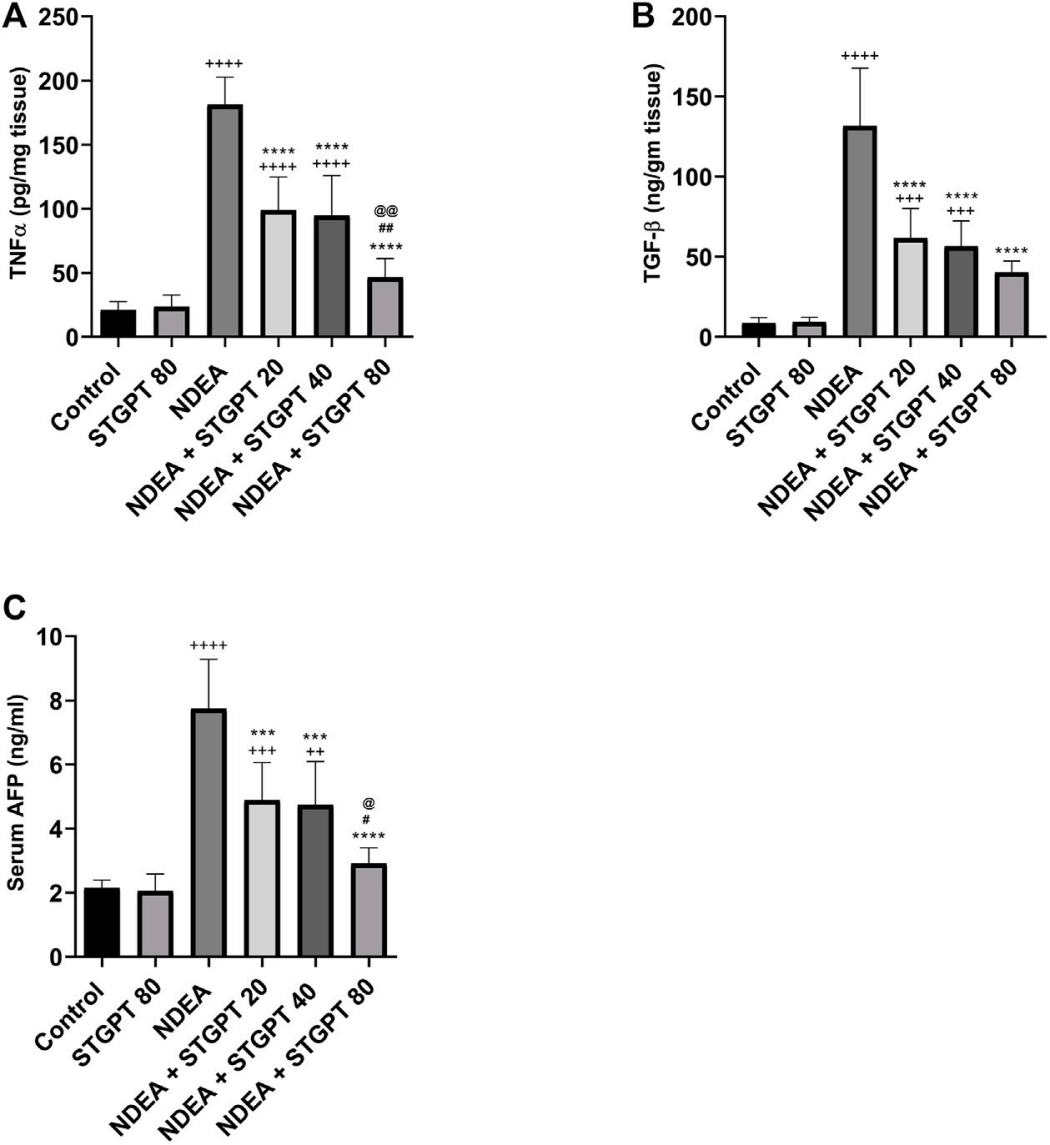
Effect of STGPT (20, 40 and 80 mg/kg) on TNF-α **(A)**; TGF-β **(B)**; and AFP **(C)** in mice with NDEA-induced HCC. Data are presented as the mean ± SD (n = 6). ^++^ *p* < 0.01 vs Control group, ^+++^ *p* < 0.001 vs Control group, ^++++^ *p* < 0.0001 vs Control group, ***p < 0.001 vs NDEA group, ****p < 0.0001 vs NDEA group, ^#^ *p* < 0.05 vs STGPT 20 group, ^##^ *p* < 0.01 vs STGPT 20 group, ^@^ *p* < 0.05 vs STGPT 40 group, ^@@^ *p* < 0.01 vs STGPT 40 group. Control, normal control group received the vehicle; STGPT 80, normal group received sitagliptin (80 mg/kg); NDEA, NDEA-induced HCC group received the vehicle; NDEA + STGPT 20, NDEA-induced HCC group treated with sitagliptin (20 mg/kg); NDEA + STGPT 40, NDEA-induced HCC group treated with sitagliptin (40 mg/kg); NDEA + STGPT 80, NDEA-induced HCC group treated with sitagliptin (80 mg/kg).

As presented in Figure 4, the administration of NDEA significantly increased the hepatic content of TNF-α, TGF-β, and the serum AFP compared to the control group. Compared to the NDEA group, treatment with STGPT 20, 40, or 80 mg/kg to the NDEA-administered mice significantly decreased the tissue content of TNF-α, TGF-β, and serum level AFP. However, treatment with STGPT 80 mg/kg was found superior to STGPT 20 and 40 mg/kg in improving TNF-α and AFP levels.

### STGPT Decreased p-AKT (Ser473)/AKT Ratio and Upregulated FOXO3 Expression in NDEA-Administered Mice

**FIGURE 5:**
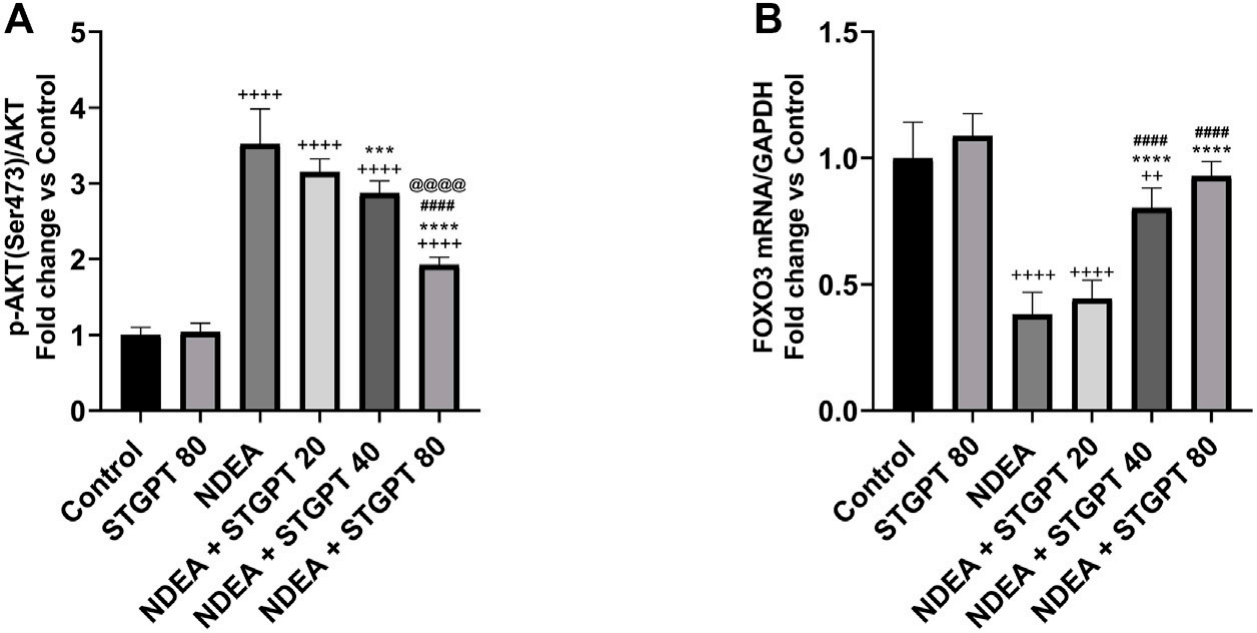
Effect of STGPT (20, 40 and 80 mg/kg) on p-AKT (ser473)/AKT **(A)**; and FOXO3 mRNA **(B)** in mice with NDEA-induced HCC. Data are presented as the mean ± SD (*n* = 6). ^++^ *p* < 0.01 *vs.* Control group, ^+++^ *p* < 0.001 *vs.* Control group, ^++++^ *p* < 0.*vs.*vs Control group, ***p < 0.*vs.* NDEA group, ****p < 0.0001 *vs.* NDEA group, ^####^ *p* < 0.*vs.* STGPT 20 group, ^@@@@^ *p* < 0.0001 *vs.* STGPT 40 group. Control, normal control group received the vehicle; STGPT 80, normal group received sitagliptin (80 mg/kg); NDEA, NDEA-induced HCC group received the vehicle; NDEA + STGPT 20, NDEA-induced HCC group treated with sitagliptin (20 mg/kg); NDEA + STGPT 40, NDEA-induced HCC group treated with sitagliptin (40 mg/kg); NDEA + STGPT 80, NDEA-induced HCC group treated with sitagliptin (80 mg/kg).

NDEA-subjected mice showed a significant increase in p-AKT (Ser473)/AKT ratio (Figure 5A) and a marked reduction in FOXO3 mRNA expression (Figure 5B) compared to the control group. Compared to the NDEA group, there was a significant reduction in the p-AKT (Ser473)/AKT ratio with concomitant upregulation in the FOXO3 mRNA after treatment with STGPT 40 80 mg/kg. While STGPT 20 mg/kg treatment resulted in a non-significant change.

### STGPT Increased p-AMPKα(Ser 487)/AMPKα, and ULK1 and Decreased p-mTOR (S2448) in NDEA-Administered Mice

**FIGURE 6:**
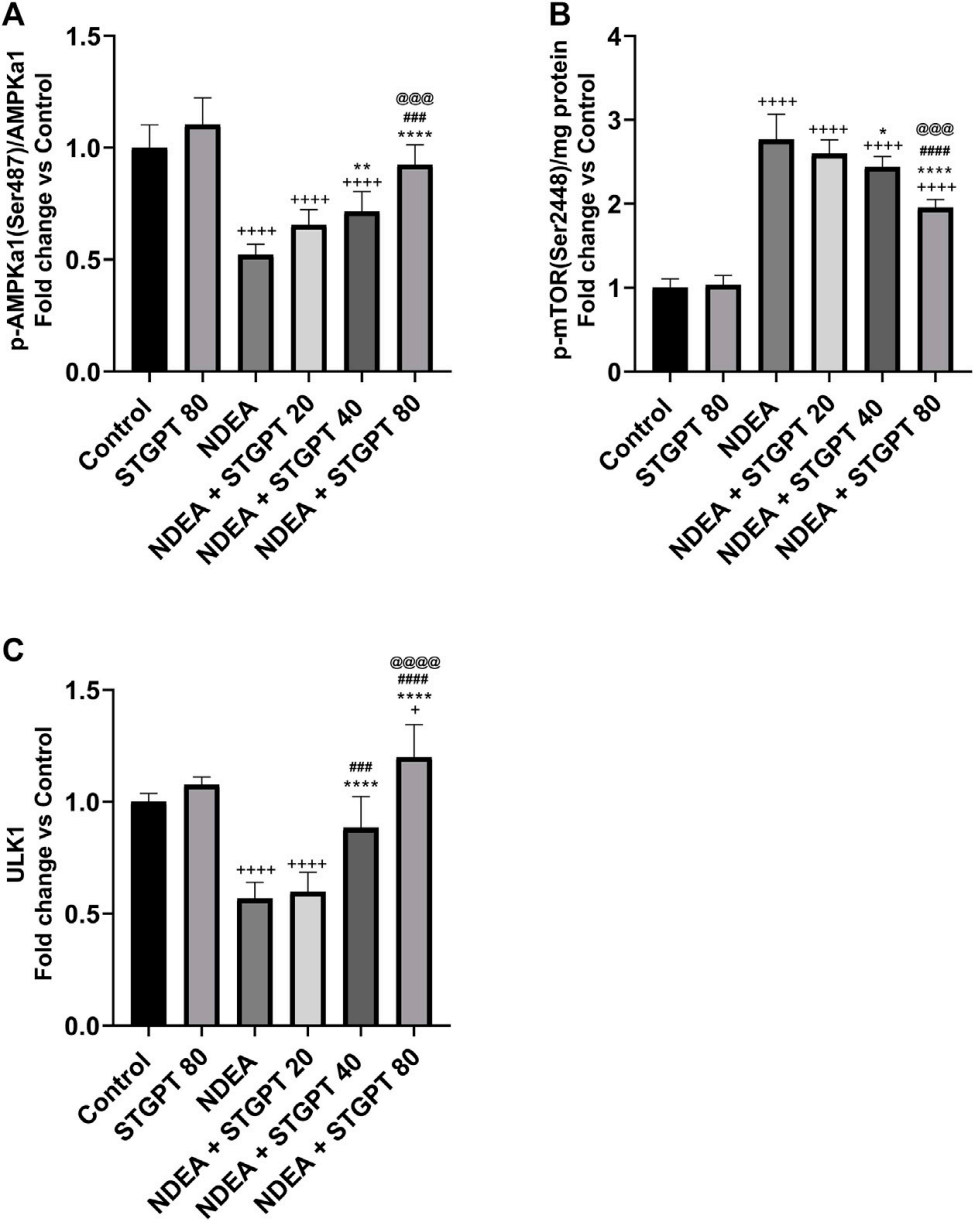
Effect of STGPT (20, 40 and 80 mg/kg) on p-AMPKα (Ser 487)/AMPKα **(A)**; p-mTOR (S2448) **(B)**; and ULK1 **(C)** in mice with NDEA-induced HCC. Data are presented as the mean ± SD (*n* = 6). ^+^ *p* < 0.05 *vs.* Control group, ^++++^ *p* < 0.0001 *vs.* Control group, *p < 0.05 vs NDEA group, **p < 0.01 *vs.* NDEA group, ****p < 0.0001 *vs.* NDEA group, ^###^ *p* < 0.001 *vs.* STGPT 20 group, ^####^ *p* < 0.0001 *vs.* STGPT 20 group, ^@@@^ *p* < 0.001 *vs.* STGPT 40 group, ^@@@@^ *p* < 0.0001 *vs.* STGPT 40 group. Control, normal control group received the vehicle; STGPT 80, normal group received sitagliptin (80 mg/kg); NDEA, NDEA-induced HCC group received the vehicle; NDEA + STGPT 20, NDEA-induced HCC group treated with sitagliptin (20 mg/kg); NDEA + STGPT 40, NDEA-induced HCC group treated with sitagliptin (40 mg/kg); NDEA + STGPT 80, NDEA-induced HCC group treated with sitagliptin (80 mg/kg).

As shown in Figure 6, mice in the NDEA group revealed a marked reduction in the p-AMPKα (Ser 487)/AMPKα ratio and ULK1 expression compared with the healthy mice in the control group. Compared to the NDEA group, these reduced levels increased significantly after treatment with STGPT 40 and 80 mg/kg, whereas the STGPT 20 group showed insignificant change. On the other hand, the level of p-mTOR (Ser 2448) in the NDEA group was significantly increased compared with the standard group. Upon treatment with STGPT in the NDEA + STGPT 40 group and NDEA + STGPT 80 group, the level of p-mTOR (S2448) was markedly decreased compared to the NDEA group. The treatment with STGPT 80 mg/kg was superior to the treatment with either STGPT 20 mg/kg or 40 mg/kg to improve these markers.

### STGPT Reduced p-p38 MAPKα, p-Ikkβ, and ERK1/2 Expression in NDEA-Administered Mice

**FIGURE 7:**
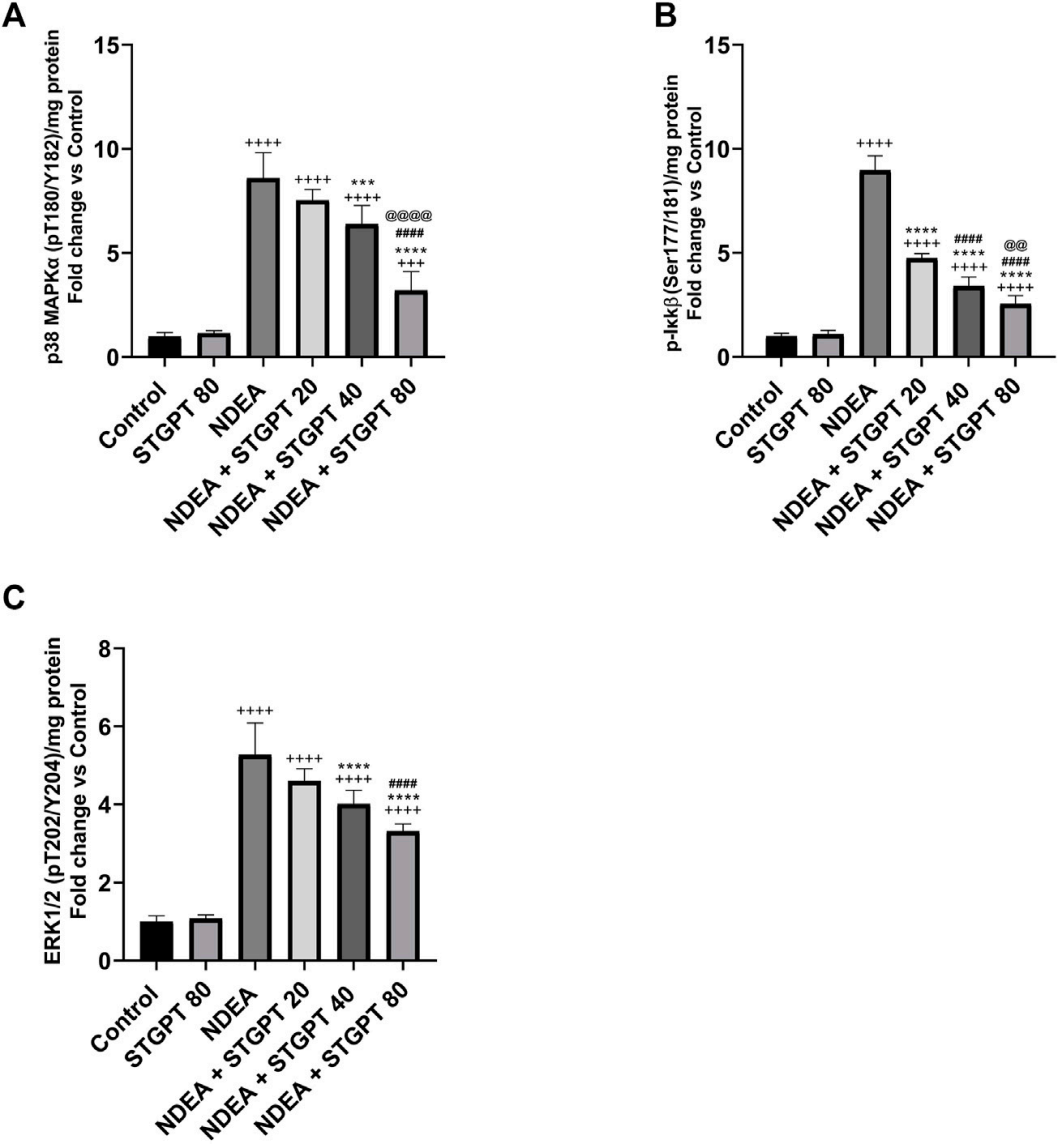
Effect of STGPT (20, 40 and 80 mg/kg) on p-p38 MAPKα **(A)**; p-Ikkβ **(B)**; and ERK1/2 **(C)** in mice with NDEA-induced HCC. Data are presented as the mean ± SD (*n* = 6). ^+++^ *p* < 0.001 *vs.* Control group, ^++++^ *p* < 0.0001 *vs.* Control group, ***p < 0.001 *vs.* NDEA group, ****p < 0.0001 *vs.* NDEA group, ^####^ *p* < 0.0001 *vs.* STGPT 20 group, ^@@^ *p* < 0.01 *vs.* STGPT 40 group, ^@@@@^ *p* < 0.0001 *vs.* STGPT 40 group. Control, normal control group received the vehicle; STGPT 80, normal group received sitagliptin (80 mg/kg); NDEA, NDEA-induced HCC group received the vehicle; NDEA + STGPT 20, NDEA-induced HCC group treated with sitagliptin (20 mg/kg); NDEA + STGPT 40, NDEA-induced HCC group treated with sitagliptin (40 mg/kg); NDEA + STGPT 80, NDEA-induced HCC group treated with sitagliptin (80 mg/kg).

Compared to a standard control, the NDEA-subjected mice showed a significant increase in p-p38 MAPKα, p-Ikkβ, and ERK1/2 (Figure 7). Compared to the NDEA group, the administration of STGPT 20, 40, or 80 mg/kg significantly decreased p-p38 MAPK and the level of p-Ikkβ; however, only the STGPT 40 mg/kg and 80 mg/kg significantly reduced the ERK1/2 levels. The 40 mg/kg dose of STGPT was more effective than the 20 mg/kg dose in reducing p-p38 MAPKα, and p-Ikkβ levels, while the 80 mg/kg dose was even more effective than the 20 and 40 mg/kg doses in reducing p-p38 MAPKα, p-Ikkβ, and ERK1/2 expressions.

### STGPT Decreased the BCL-2: Bax Ratio and Ki-67 mRNA and Increased p53 mRNA Expression in NDEA-Administered Mice

**FIGURE 8:**
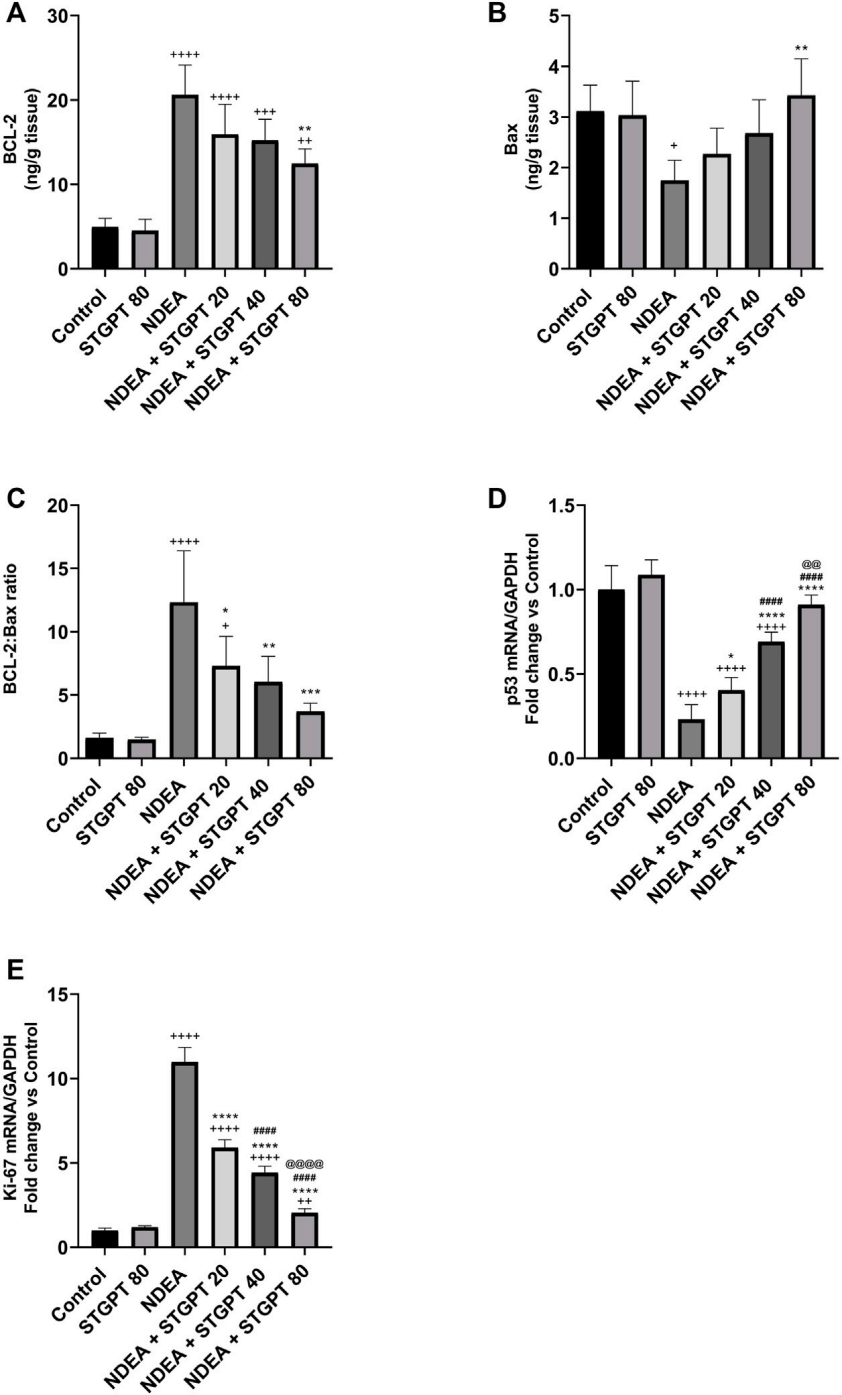
Effect of STGPT (20, 40 and 80 mg/kg) on BCL-2 **(A)**; Bax **(B)**; BCL-2: Bax ratio **(C)**; p53 mRNA **(D)**; and Ki-67 mRNA **(E)** in mice with NDEA-induced HCC. Data are presented as the mean ± SD (*n* = 6). ^+^ *p* < 0.05 vs Control group, ^++^ *p* < 0.01 *vs.* Control group, ^+++^ *p* < 0.001 *vs.* Control group, ^++++^ *p* < 0.0001 *vs.* Control group, *p < 0.05 *vs.* NDEA group, **p < 0.01 *vs.* NDEA group, ***p < 0.001 *vs.* NDEA group, ****p < 0.0001 *vs.* NDEA group, ^####^ *p* < 0.0001 *vs.* STGPT 20 group, ^@@^ *p* < 0.01 *vs.* STGPT 40 group, ^@@@@^ *p* < 0.0001 *vs.* STGPT 40 group. Control, normal control group received the vehicle; STGPT 80, normal group received sitagliptin (80 mg/kg); NDEA, NDEA-induced HCC group received the vehicle; NDEA + STGPT 20, NDEA-induced HCC group treated with sitagliptin (20 mg/kg); NDEA + STGPT 40, NDEA-induced HCC group treated with sitagliptin (40 mg/kg); NDEA + STGPT 80, NDEA-induced HCC group treated with sitagliptin (80 mg/kg).

In comparison to the control group, mice with NDEA-induced HCC had a significant increase in BCL-2 (Figure 8A) and a significant decrease in Bax content (Figure 8B), resulting in a significant rise in the BCL-2: Bax ratio (Figure 8C). Only STGPT 80 mg/kg caused a significant improvement in BCL-2 and Bax content, but all STGPT-treated groups showed a significant reduction in the BCL-2: Bax ratio compared to the NDEA group. The NDEA-subjected mice showed a significant decrease in the hepatic expression of p53 (Figure 8D) with a concomitant upregulation of Ki-67 mRNA (Figure 8E) compared to the standard control. Compared to the NDEA group, STGPT 20, 40, or 80 mg/kg administration to NDEA-administered mice significantly increased the mRNA expression of p53. It decreased the Ki-67 mRNA expression when compared to the NDEA group. Treatment with STGPT 80 mg/kg was found superior to STGPT 20 and 40 mg/kg in improving p53 levels. The 40 mg/kg dose was more effective than the 20 mg/kg dose in reducing Ki-67 mRNA expression, while the 80 mg/kg dose was even more effective than the 20 and 40 mg/kg doses in reducing Ki-67 mRNA expression.

### STGPT Increased the TIMP-1/MMP-2 Ratio and Decreased VEGF, CD309 mRNA, and HIF-1α in NDEA-Administered Mice

**FIGURE 9:**
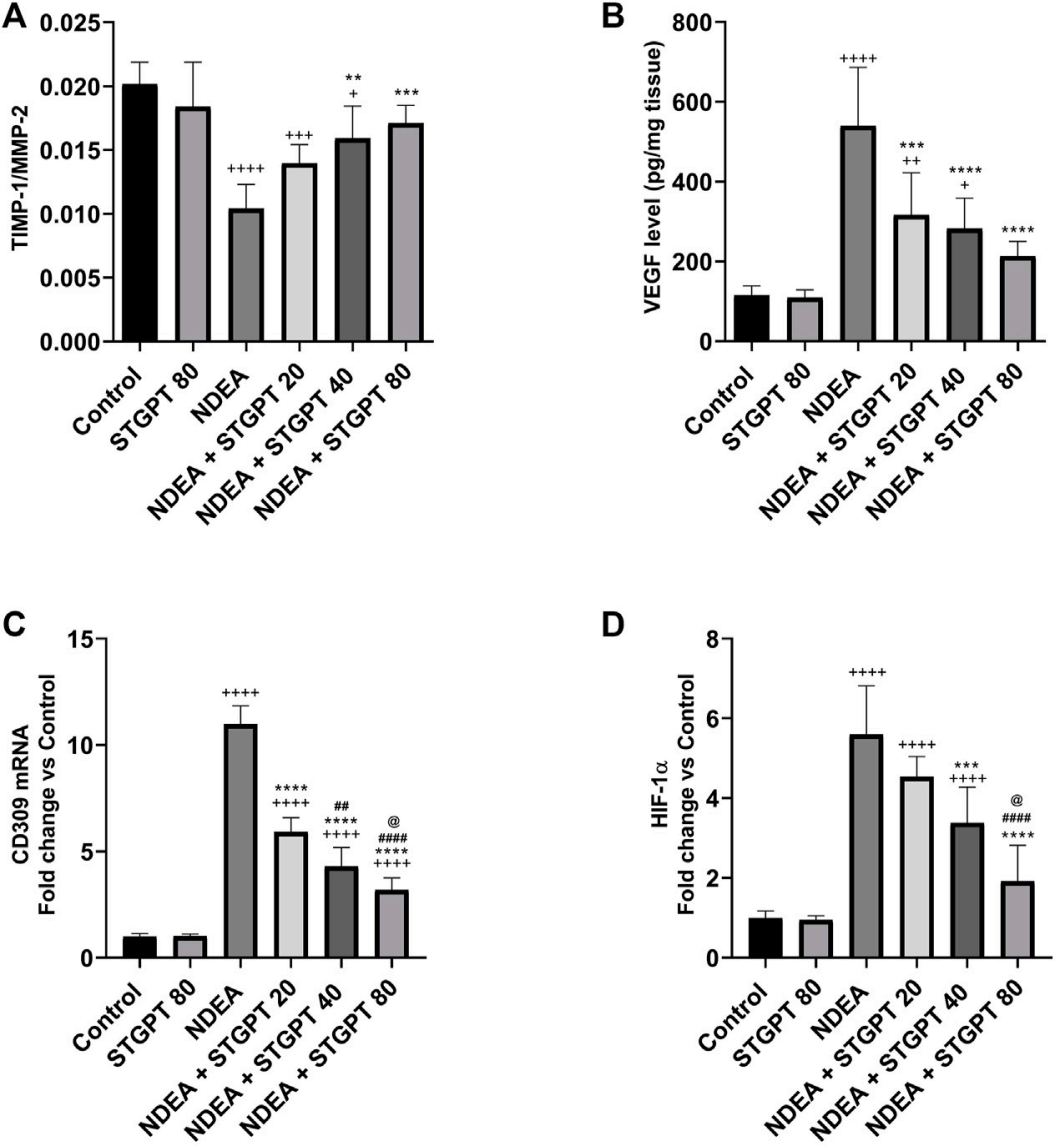
Effect of STGPT (20, 40 and 80 mg/kg) on TIMP-1/MMP-2 ratio **(A)**; VEGF **(B)**; CD309 mRNA **(C)**; and HIF1-α **(D)** in mice with NDEA-induced HCC. Data are presented as the mean ± SD (*n* = 6). ^+^ *p* < 0.05 *vs.* Control group, ^++^ *p* < 0.01 *vs.* Control group, ^+++^ *p* < 0.001 *vs* Control group, ^++++^ *p* < 0.0001 vs Control group, **p < 0.01 vs NDEA group, ***p < 0.001 *vs.* NDEA group, ****p < 0.0001 *vs.* NDEA group, ^##^ *p* < 0.01 vs STGPT 20 group, ^####^ *p* < 0.0001 *vs.* STGPT 20 group, ^@^ *p* < 0.05 *vs.* STGPT 40 group. Control, normal control group received the vehicle; STGPT 80, normal group received sitagliptin (80 mg/kg); NDEA, NDEA-induced HCC group received the vehicle; NDEA + STGPT 20, NDEA-induced HCC group treated with sitagliptin (20 mg/kg); NDEA + STGPT 40, NDEA-induced HCC group treated with sitagliptin (40 mg/kg); NDEA + STGPT 80, NDEA-induced HCC group treated with sitagliptin (80 mg/kg).

Mice with NDEA-induced HCC showed a marked decrease in the TIMP-1/MMP-2 ratio (Figure 9A) compared with the standard control group. Following the administration of STGPT 40 or 80 mg/kg, a significant increase in the TIMP-1/MMP-2 ratio was observed, whereas the 20 mg/kg did not produce a significant increase compared with the NDEA group. The tissue content of VEGF (Figure 9B), HIF-1α (Figure 9D), and the mRNA expression of CD309 (Figure 9C) were significantly increased in the NDEA group when compared to the control group. Upon treatment with the STGPT 20, 40, or 80 mg/kg, a significant reduction in hepatic content of VEGF and the mRNA expression of CD309 was observed when compared to the NDEA group. While the treatment with STGPT 40 or 80 mg/kg significantly reduced HIF-1α, the treatment with STGPT 20 mg/kg resulted in a non-significant reduction compared with the NDEA group. Treatment with STGPT 80 mg/kg was superior to STGPT 20 and 40 mg/kg in improving HIF-1-α levels.

### STGPT Downregulated MCP-1 mRNA and did Not Affect GLP-1R mRNA in NDEA-Administered Mice

**FIGURE 10:**
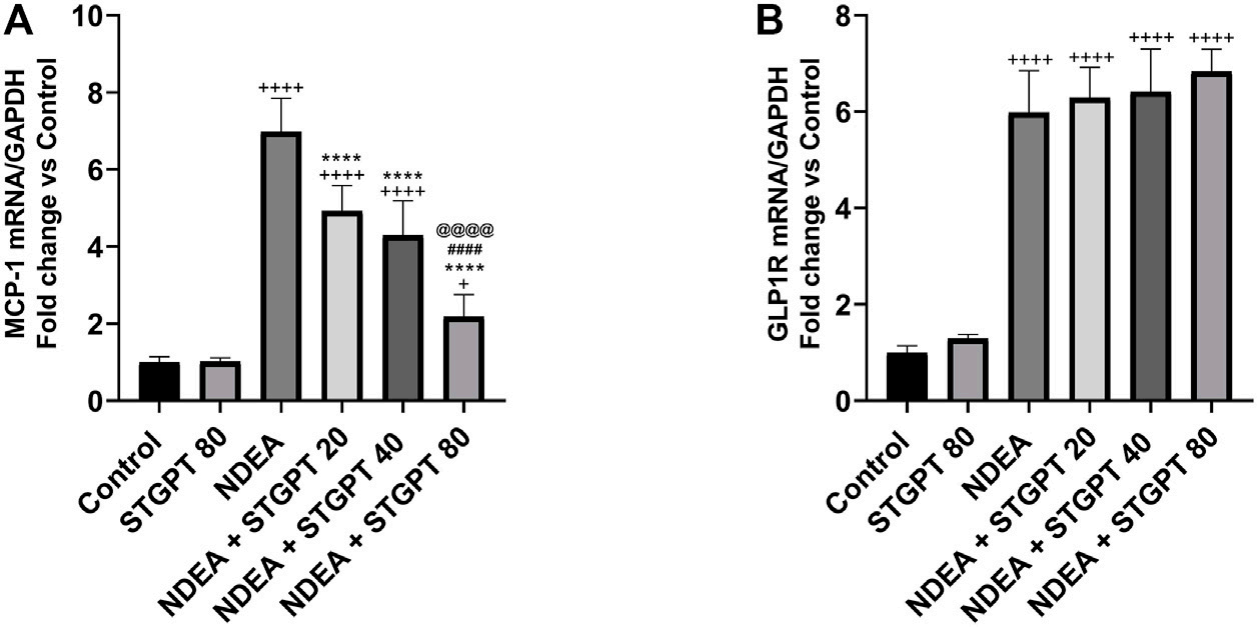
Effect of STGPT (20, 40 and 80 mg/kg) on MCP-1 mRNA **(A)** and GLP-1R mRNA **(B)** in mice with NDEA-induced HCC. Data are presented as the mean ± SD (*n* = 6). ^+^ *p* < 0.05 *vs.* Control group, ^++++^ *p* < 0.0001 *vs.* Control group, **p < 0.01 *vs.* NDEA group, ****p < 0.0001 *vs.* NDEA group, ^####^ *p* < 0.0001 *vs.* STGPT 20 group, ^@@@@^ *p* < 0.0001 *vs.* STGPT 40 group. Control, normal control group received the vehicle; STGPT 80, normal group received sitagliptin (80 mg/kg); NDEA, NDEA-induced HCC group received the vehicle; NDEA + STGPT 20, NDEA-induced HCC group treated with sitagliptin (20 mg/kg); NDEA + STGPT 40, NDEA-induced HCC group treated with sitagliptin (40 mg/kg); NDEA + STGPT 80, NDEA-induced HCC group treated with sitagliptin (80 mg/kg).

Compared to the control group, NDEA administration resulted in a significant upregulation of the MCP-1 mRNA (Figure 10A). Treatment with STGPT 20, 40, or 80 mg/kg, on the other hand, significantly downregulated this level when compared to the NDEA group. The 80 mg/kg dose of STGPT was superior to 20 and 40 mg/kg in decreasing the MCP-1 mRNA expression. The GLP-1R mRNA was significantly upregulated in the NDEA group compared to the control group (Figure 10B). It is worth noting that none of the used STGPT doses resulted in a significant change in the expression of GLP-1R compared to the NDEA group.

## Discussion

HCC remains a deadly disease with a poor prognosis in patients with unresectable cancer (Ko et al., 2020). Current chemotherapies have a lot of adverse effects, such as bone marrow suppression, oral mucositis, and neurotoxicity (Nurgali et al., 2018). Previous studies have shown that DM has a significant impact on the progression of HCC (El-Serag et al., 2001; Huang et al., 2017; Yang et al., 2020). In the present study, we hypothesized that using STGPT, an antidiabetic drug, could provide a new therapeutic modality for managing HCC. In the present study, dysplastic nodules were not visible on gross examination of harvested livers. However, distinct atypia of the large liver cell dysplasia characterizes dysplastic lesions and preneoplastic changes. The present study revealed that treatment using STGPT (80 mg/kg) improved liver function, histological characteristics and increased the survival rate compared with those of the NDEA group of rats.

The parenteral or oral exposure to small quantities of NDEA results in liver damage and eventually hepatocarcinogenesis which corresponds to human HCC (El-Ashmawy et al., 2016; El-Ashmawy et al., 2017). In the present study, the NDEA group showed enlarged livers with a greater liver index indicating that NDEA increased the proliferation of hepatic cells supported by the increase in the necroinflammation score, the upregulation of Ki-67 mRNA expression, and AFP level. Ki-67 is a nuclear protein and is considered one of the major inducing factors of tumor proliferation (Luo et al., 2015). Serum AFP levels showed good accuracy in HCC diagnosis and have been proved to act as an independent risk predictor associated with progression and survival (Bai et al., 2017). The present findings agree with those of Heindryckx et al. (2009), who reported that NDEA induced neoplastic changes were associated with a significant increase in liver function tests, liver size, and liver index. NDEA was reported to increase oxidative stress (Moreira et al., 2015); in the present study, we found that NDEA increased oxidative stress as indicated by the decrease in the TAC and the upregulation of the MCP-1 mRNA, which is known to be stimulated by oxidative stress. Dagouassat et al. (2010) found that myofibroblast-derived MCP-1 could be involved in the pathogenesis of HCC. Additionally, MCP-1 recruits monocytes, memory T cells, and dendritic cells to the sites of inflammation produced by tissue injury. Therefore, together with its effect on TNF-α, STGPT exhibited a considerable anti-inflammatory effect.

The present findings were supported by the inhibitory effect of STGPT on TGF-β, an inducer of liver proliferation and hepatocarcinogenesis (Ren et al., 2019). STGPT at different doses exerted its action through decreasing oxidative stress, as illustrated by the increase in TAC and downregulation of MCP-1 (Kabel et al., 2018).

Hypoxia is caused by defective tissue vascularization and poor blood supply, and it is an established characteristic of all solid tumors. HIF-1, a heterodimeric transcriptional factor composed of HIF-1*α* and HIF-1*β* subunits, has a crucial role in maintaining oxygen homeostasis (El-Gizawy et al., 2020). In hypoxic conditions, HIF-1*α* induces tumor angiogenesis, proliferation, metastasis and inhibits apoptosis of cancer cells. In HCC, tumor hypoxia worsens the patient’s prognosis (Luo et al., 2014). In the present study, NDEA-administered mice showed an increase in HIF-1*α* compared to the regular control group, which may also be attributed to the increase in TGF-β level (Mallikarjuna et al., 2019). TGF-β1 increased HIF-1α levels in both normoxic and hypoxic conditions, which correlate to the present findings (Basu et al., 2011). Besides, NDEA treatment also afforded commendable hypoxia, which was perceived via boosting the HIF-1α, mediated by reducing PHD2 expression (Cui et al., 2018). On the other hand, treatment with STGPT in different groups decreased HIF-1*α* levels compared to that in the NDEA group. STGPT reduced HIF-1α levels in testicular torsion/detorsion in rats, which correspond with the present results (Abdelzaher et al., 2020). The HIF-1*α* decrease found in STGPT-treated groups may be attributed to different mechanisms.

The first proposed mechanism for the inhibition of HIF-1*α* is through the activation of AMPK. AMPK, a serine/threonine kinase expressed in many tissues, is an essential mediator in maintaining cellular energy homeostasis. AMPK is emerging as a possible metabolic tumor suppressor and target for cancer prevention and treatment (Li et al., 2015). In the present study, NDEA induces inhibition of AMPK and thus decreases phosphorylation of AMPK levels (Lee et al., 2019). Treatment with STGPT, on the other hand, induced phosphorylation and consequently activated AMPK (Balteau et al., 2014).

AMPK activation by STGPT induced autophagy by activating ULK1 at Ser317, thereby decreasing tumor cell survival (Alers et al., 2012). STGPT activation of AMPK inhibits mTORC1 activity and tuberous sclerosis protein 2 (TSC2) (Shaw, 2009 **)**. mTOR is a serine/threonine-protein kinase that regulates cell growth, cell proliferation, and cell survival. AMPK inhibits mTOR through the phosphorylation of TSC2. Therefore, STGPT, through activation of AMPK, stimulated ULK1 and inhibited mTOR, which in turn inhibits HIF.

The second mechanism through which STGPT treatment groups might inhibit HIF is the inhibition of AKT that stimulates apoptosis, as revealed by a decreased BCL-2/Bax ratio and an increased P53 gene expression. AKT, a serine/threonine kinase, is an oncogenic protein that regulates cell survival, proliferation, growth, apoptosis, and glycogen metabolism. Active AKT stimulates anti-apoptotic Bcl-2 while inhibiting pro-apoptotic Bax protein and P53 levels and thus increased Bcl-2/Bax ratio (Lires-Deán et al., 2008; Akl et al., 2014). The current study showed a significant increase in the AKT level in the NDEA-administered mice. In contrast, the treatment of mice with different STGPT doses decreased AKT level, which may also be attributed to the decreased TGF-β level (Hamidi et al., 2017). The reversal of those findings after treatment using different STGPT doses decreased the survival of cancer cells and increased the survival rate of the animals.

The present results showed that inhibition of AKT using STGPT upregulated FOXO gene expression. This AKT downstream substrate leads to tumor suppression and thus increases the survival rate in all STGPT treated groups (Zhang and Chen, 2019).

The third mechanism through which HIF might be downregulated in STGPT treated groups is through control of MAPK. MAPK, serine/threonine kinases regulate cell proliferation, differentiation, and survival. The MAPK family has three subtypes: JNK1/2/3, ERK, and p38 MAPK. MAPK signaling upregulation has been implicated in HIF activation (Sang et al., 2003).

The present findings demonstrate that NDEA increased ERK and p38 MAPK, which may explain that TGF-β activates all ERK and p38 MAPK in numerous cell types through Smad dependent and independent mechanisms with subsequent mechanisms of HIF stimulation. On the other hand, STGPT decreased ERK and p38 MAPK partially explained by its inhibitory effect on TGF-β (Shi et al., 2017; Al-Damry et al., 2018).

The previously mentioned factors for the increased HIF level in the NDEA group led to changes in epithelial and mesenchymal transition and angiogenesis, which worsened the case (Tam et al., 2020). In the present study, NDEA administration decreased the TIMP-1/MMP-2 ratio. At the same time, increased VEGF and upregulated the expression of its receptor, CD309, indicating an increase in angiogenesis, metastasis, and epithelial and mesenchymal transition (EMT), which the increase in HIF level may interpret (Okuyama et al., 2006; Wan et al., 2011; Hatfield et al., 2014). On the other hand, STGPT-treated groups showed an increase in TIMP-1/MMP-2 while decreasing VEGF and downregulating the expression of CD309, indicating a decrease in angiogenesis, metastasis, and EMT, which may be interpreted by the decrease in HIF level (Yang et al., 2017; Kosowska and Garczorz, 2020 **)**.

## Conclusion

STGPT repressed N-nitrosodiethylamine-induced murine hepatocellular carcinoma progression by inhibiting HIF-1α via the interference with the AKT-AMPKα-mTOR axis and the interruption of IKKβ, P38α, and ERK1/2 signals as well (Supplementary Material). Accordingly, STGPT relieved oxidative stress, inhibited angiogenesis and tissue invasion. Additionally, STGPT exhibited apoptotic stimulatory effect and antiproliferative activity. These effects appear to be independent of GLP1R as STGPT did not significantly alter the mRNA expression of GLP1R in the context of HCC progression. However, further investigations are compulsory. The present study provides a potential basis for repurposing STGPT for the inhibition of HCC progression. Since sitagliptin is unlikely to cause hypoglycemia, it may be promising as monotherapy or adjuvant therapy for treating diabetic or even normoglycemic patients with HCC.

## Data Availability

The raw data supporting the conclusions of this article will be made available by the authors, without undue reservation.

## Abbreviations

AKT, protein kinase B; AMPK, 5′ AMP-activated protein kinase; Bax, BCL2-associated X protein; BCL-2, B-cell lymphoma 2; FOXO3, forkhead box O3; GLP1R, glucagon-like peptide-1 receptor; IκBκB, the inhibitor κB kinase complex; HIF-1α, hypoxia-inducible factor 1-alpha; MAPK, mitogen-activated protein kinase; MCP-1, monocyte chemoattractant protein-1; MMP-2, matrix metalloproteinase-2; mTOR, mechanistic target of rapamycin; TIMP-1, tissue inhibitor of metalloproteinase-1; TNF-α, tumor necrosis factor-alpha; VEGF, vascular endothelial growth factor; AFP, alpha-fetoprotein; ULK1, UNC-51-like kinase 1.
