# Supplementary figures and images for: AKT-AMPKα-mTOR-dependent HIF-1α Activation is a New Therapeutic Target for Cancer Treatment: A Novel Approach to Repositioning the Antidiabetic Drug Sitagliptin for the Management of Hepatocellular Carcinoma
Source: Front Pharmacol. 2022 Jan 12;12:720173. doi: 10.3389/fphar.2021.720173 (PMC8790251; doi:10.3389/fphar.2021.720173)

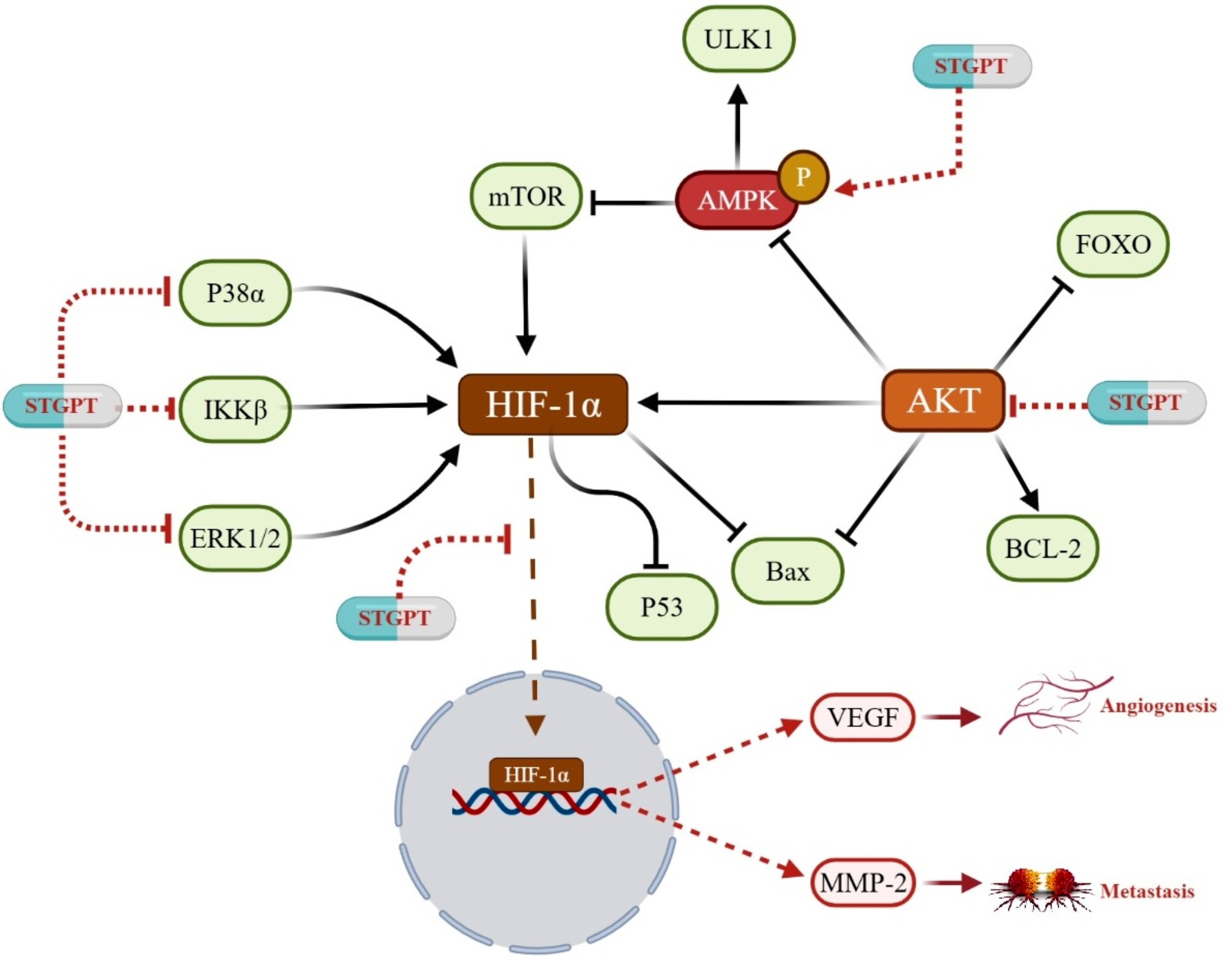

Supplement: Supplementary file 1 [file Image1.JPEG]
